# Craving More Than Meals: Social Isolation Among Older Adults on Meals on Wheels Waitlists

**DOI:** 10.1093/geroni/igaf122.478

**Published:** 2025-12-31

**Authors:** Kimberly Bernard, Emily Gadbois, Melissa Clark, Em Balkan, Laura Dionne, Christopher Liu, Kali Thomas

**Affiliations:** Brown University, Providence, Rhode Island, United States; Brown University School of Public Health, Providence, Rhode Island, United States; Brown University, Providence, Rhode Island, United States; Brown University, Providence, Rhode Island, United States; Brown University, Providence, Rhode Island, United States; Brown University, Providence, Rhode Island, United States; Johns Hopkins University, Baltimore, Maryland, United States

## Abstract

Prolonged periods of social isolation are increasingly found to have negative impacts on health and well-being. Older adults are particularly at risk as social networks tend to decrease after 65 years of age. Recent nationally representative studies have found that 1 in 4 community dwelling older adults are socially isolated. Slightly more than half of the participants in the Deliver-EE clinical trial lived alone (54%). Approximately, 20% of the total sample reported that they often felt isolated from others, this percent was similar between those who lived alone and lived with others. We examined whether those experiencing social isolation were more likely to request meal schedules that provided regular contact with a paid/volunteer driver five times per week versus an alternative (once every two weeks). We found that those who were lonely were more likely to request daily deliveries (45% versus 41%). To better understand the potential impacts of delivery contacts on socially isolated participants, we analyzed 54 qualitative client interviews for reports of interactions with delivery drivers. Clients described a range of socialization experiences from “brief and polite” to having more substantive conversations with their drivers. When probed, clients often shared that they will refrain from initiating conversation out of concern that they will put the driver off their schedule. With active encouragement, socially isolated older adults could get additional benefits from meal programs that provide daily delivery touch points.

